# PD-L1 Expression in Mastocytosis

**DOI:** 10.3390/ijms20092362

**Published:** 2019-05-13

**Authors:** Margaret Williams, Diane S. Lidke, Karin Hartmann, Tracy I. George

**Affiliations:** 1 Department of Pathology, University of Utah, Salt Lake City, UT 84112, USA; margaret.williams@hsc.utah.edu; 2 ARUP Laboratories, Salt Lake City, UT 84108, USA; 3 Department of Pathology and Comprehensive Cancer Center, University of New Mexico, Albuquerque, NM 87102, USA; Dlidke@salud.unm.edu; 4 Division of Allergy, Department of Dermatology, University of Basel, 4031 Basel, Switzerland; karin.hartmann@usb.ch

**Keywords:** immune checkpoint, PD-1, PD-L1, mastocytosis

## Abstract

Programmed death 1 (PD-1), when activated by its ligands PD-L1 and PD-L2, suppresses active immune cells in normal immune regulation to limit autoimmunity and, in tumors, as a mechanism of immune evasion. PD-L1 expression has been described as both a prognostic and predictive marker in many solid and hematologic neoplasms, as targeted therapies against the PD-1/PD-L1 interaction have gained clinical importance. PD-L1 expression has been assessed in a few studies on mastocytosis. We review this literature and the need for further investigation of the tumor-immune interaction in mastocytosis.

## 1. Immune Checkpoint Inhibitors in Cancer

Programmed death 1 (PD-1), first described in 1992, has been described on many activated immune cells, including both CD4 T-cells, CD8 T-cells, B-cells, NK/T cells, dendritic cells and macrophages. When PD-1 binds its ligands, PD-L1 and PD-L2, active immune cells are inhibited as an important check on protecting tissues from autoimmunity [1]. PD-L1 expression has been shown to be upregulated in many tumor types as a mechanism of immune suppression and evasion [1]. 

PD-L1 expression has been assessed in many solid and hematologic malignancies, both as a prognostic marker and as a predictive marker of the response to antibodies that block the PD-1/PD-L1 interaction [1,2,3,4,5]. PD-L1 expression as a predictive biomarker for therapy has become routine clinical practice in several solid malignancies [3,4,5,6]. Since the first U.S. Food Drug Administration (FDA) approved antibody targeting the PD-1/PD-L1 interaction was registered in 2011 in melanoma, immune checkpoint inhibitors have become increasingly important in clinical use. In 2018, the Nobel Prize in medicine was awarded to James Allison and Tsauku Honjo for their discovery of the inhibition of negative immune regulation in cancer therapy [1]. In hematologic malignancies, pembrolizumab is now an FDA-approved therapy for classic Hodgkin lymphoma, and clinical trials including various checkpoint inhibitors have been performed on multiple myeloma, chronic lymphocytic leukemia, acute myeloid leukemia, myelodysplastic syndrome, diffuse large B-cell lymphoma, follicular lymphoma, and cutaneous T-cell lymphoma [2]. 

An immunohistochemical (IHC) assessment of PD-L1 expression has been used to predict the response to and qualify patients for immune checkpoint inhibitors [7]. In some tumors, the clinical response to immune checkpoint therapies correlates with PD-L1 positivity within tumor cells; however, in other tumors a clinical response has also been shown in patients with tumors with a low PD-L1 expression. For example, in ongoing clinical trials in hepatocellular carcinoma, a clinical response has been seen with PD-L1 inhibition without a clear predictive role of the PD-L1 expression, with an accelerated FDA approval for nivolumab as a second line therapy without restriction by the PD-L1 status [8,9]. In other tumor types, immune checkpoint therapies are approved as part of combination therapies, regardless of the PD-L1 expression, as exemplified by the current National Comprehensive Cancer Network guidelines, including the use of pembrolizumab as part of combination therapies in certain patients with non-small cell lung carcinoma [7,10].

Because PD-L1 expression in tumor cells represents a mechanism of immune evasion via the interaction between the PD-L1 ligands on tumor cells and PD-1 on lymphocytes to suppress the immune response, an assessment of PD-1 within tumor-infiltrating lymphocytes is important for understanding the tumor-immune interaction. In melanoma, previous studies have examined the role of both innate and adaptive immunity, where PD-L1 expression has been proposed in some cases to be driven by oncogenic mutations, and in others, to be mediated by cytokines [11]. 

## 2. PD-1 and PD-L1 Expression in Mastocytosis

While patients with cutaneous mastocytosis (CM) and indolent systemic mastocytosis (ISM) generally do not require anti-neoplastic therapy, current therapeutic options in advanced systemic mastocytosis (advSM) include cladribine, midostaurin, imatinib, and hematopoietic stem cell transplants [12]. Multiple additional tyrosine kinase inhibitors are currently being investigated in clinical trials [12]. There are no current clinical trials assessing the use of immune checkpoint inhibitors in mastocytosis, but a few groups have published on the expression of PD-1 and PD-L1 in mastocytosis, as summarized in Table 1.

In 2013, Kataoka et al. assessed the PD-1 expression by IHC within skin biopsies from patients with CM compared to patients with non-specific dermatitis and demonstrated PD-1 positivity within 10/30 CM cases and no PD-1 staining within the non-specific dermatitis cases [13]. A limitation of interpreting the presence of PD-1 within the patient samples is the absence of clinical data to assess whether the skin biopsies represented a limited skin involvement by CM or cutaneous involvement secondary to systemic mastocytosis (SM). The authors further assessed PD-1 expression within cultured mast cell lines by flow cytometry and reverse-transcriptase polymerase chain reaction (RT-PCR), demonstrating PD-1 mRNA within the LAD2 cell line, a human mastocytosis cell line with wildtype KIT, while no PD-1 mRNA was detected within the HMC1.2 cell line, a human mastocytosis cell line with V560G and D816V *KIT* mutations. Following stimulation of the mast cell lines with recombinant PD-L1, there was suppressed growth within the LAD2 cell line. 

In 2016, Kuklinski et al. assessed the PD-L1 expression within 16 skin biopsies of patients with diverse mastocytosis types, including diffuse CM, solitary mastocytoma, and SM involvement (mastocytosis in skin) and found strong and diffuse PD-L1 staining within all mastocytosis specimens, regardless of the subtype [14]. There was little to no staining for PD-L1 within normal skin biopsies. 

Additionally, in 2016, Rabenhorst et al. examined PD-1, PD-L1, PD-L2, and tryptase within the serum of 43 patients with mastocytosis and 22 healthy controls, demonstrating significantly increased PD-L1 within the serum of adult patients with mastocytosis, compared to controls. In addition, serum PD-L1 was significantly elevated in patients with aggressive SM (ASM), SM with an associated hematologic neoplasm (SM-AHN), and mast cell leukemia (MCL), compared to patients with a non-advanced disease [15]. They found no difference between PDL-2 in the serum of adult patients with mastocytosis and the controls. Interestingly, they identified significantly higher levels of PD-L1 and PD-1 in the serum of normal children compared to normal adults and no significant difference between PD-L1 or PD-1 in children with mastocytosis compared to pediatric controls. As expected epidemiologically, all of the pediatric patients with mastocytosis had CM. In addition to the evaluation of these markers in the serum, PD-1, PD-L1, PD-L2 and tryptase were evaluated by immunofluorescence in skin and bone marrow biopsies of patients with mastocytosis. While PD-L1 expression was observed co-localizing with tryptase within both the skin and bone marrow, PD-1 expression was only observed in the cutaneous mast cells.

**Figure 1:**
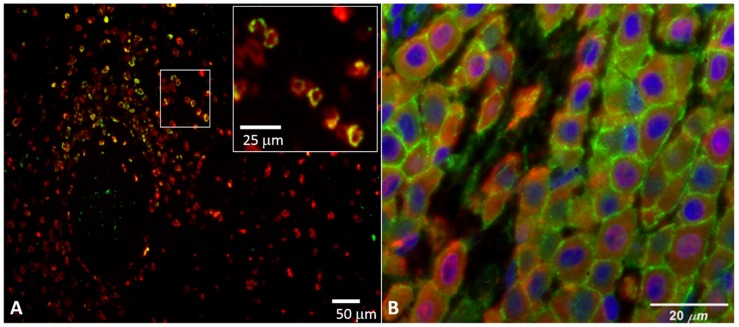
PD-L1 staining in neoplastic mast cells using multicolor immunohistofluorescence (see methods in Hatch et al., 2018 [16]): (**A**) spleen tissue from a patient with mast cell leukemia, stained for mast cells (tryptase, red) and programmed death-ligand 1(PD-L1) (light green); (**B**) skin from a patient with cutaneous mastocytosis, stained for mast cells (tryptase, red), PD-L1 (light green), and nuclei (4’,6-diamidino-2-phenylindole (DAPI), blue).

In 2018, Hatch et al examined PD-1 and PD-L1 by IHC in 55 tissues from patients with mastocytosis, demonstrating a PD-L1 expression in 77% of bone marrow biopsies and 92% of skin biopsies including all mastocytosis types; they found no expression of PD-1 or PD-L1 within mast cells in healthy or reactive bone marrows or cases of myelodysplastic syndrome or myeloproliferative neoplasms [16]. PD-L1 surface expression was confirmed by flow cytometry in patients with ISM and ASM. PD-1 expression was identified in 15% of CM cases, and was not identified in the skin lesions of patients with SM. Correlative studies using multicolor immunohistofluorescence (IHF) showed that the PD-L1 expression in mastocytosis was heterogeneous, with only a subset of mast cells expressing PD-L1 in MCL (Figure 1A), while nearly all mast cells in CM were positive for PD-L1 (Figure 1B). They also demonstrated an architectural variation in the PD-L1 positivity within the spleen of a patient with MCL, where PD-L1-expressing mast cells were often found near the periarteriolar lymphatic sheaths.

## 3. Conclusions

Several groups have demonstrated PD-L1 expression in neoplastic mast cells. While PD-1 expression was present in a subset of CM cases reported by both Katoaka et al. and Hatch et al., interestingly, PD-L1 expression was limited to neoplastic mast cells and was not reported in tumor infiltrating lymphocytes [13,16]. The presence of a T-cell lymphocytic infiltrate in association with the neoplastic mast cell infiltrates in the spleen may suggest a cytokine-mediated mechanism of the PD-L1 expression, which could be consistent with the variability of the PD-L1 expression identified within neoplastic mast cells, even within the same tissue in a given patient [16]. In cases with no identifiable or limited T-cell infiltrates, an underlying genetic or epigenetic mutation may underlie an increased PD-L1 expression by the neoplastic mast cells. Interestingly, in the bone marrow of patients with SM, lymphoid infiltrates are seen more frequently in indolent SM, whereas the bone marrow from patients with advSM typically lacks significant lymphocytic infiltrates (personal observations, T.I.G). This may have implications for the efficacy of immune checkpoint therapies both between patients and within different tissues in a single patient [16].

In the Rabenhorst et al. 2016 cohort, there was a breakpoint with significantly higher PD-L1 in the serum of adult patients with advSM (ASM, SM-AHN, and MCL), compared to non-advanced mastocytosis (ISM and CM). There was no clear difference in the intensity of the staining or the percentage of the mast cells staining for PD-L1 by mastocytosis subtype by IHC in the Hatch et al. cases. While the prognostic and predictive value of PD-L1 expression within tumor tissue is well-established, the clinical utility of serum PD-L1 measurements is under investigation and is still unknown, limited by a lack of robust data [17].

In addition to the difference in the PD-L1 detection by mastocytosis subtype in serum versus by IHC on tissue biopsies, there was a variability seen in the percentage of mast cells staining for PD-L1 within the same splenic tissue reported in Hatch et al. This variability of staining within the same tissue has implications for the use of PD-L1 as a potential prognostic or predictive biomarker. Future studies could include both a serum and tissue assessment of PD-L1 with an assessment of PD-L1 in multiple tissues within the same patient, in order to better characterize the expression within different types of mastocytosis and the variability in the staining patterns. The PD-L1 expression in neoplastic mast cells that has been confirmed by these studies warrants further investigation of the tumor-immune interactions in mastocytosis to clarify possible breakpoints in the PD-L1 expression by type of mastocytosis, as well as to clarify the role of the immune checkpoint molecule expression in tumor infiltrating lymphocytes, and to explore a possible role for immune checkpoint inhibition.

## Figures and Tables

**Table 1:** Studies Examining PD-L1 Expression in Mastocytosis.

Author	Method	Major Findings
Kataoka et al.	IHC on skin biopsies, flow cytometry and RT-PCR on mast cell lines	PD-1 expressed in 33% (*n* = 30) of skin biopsies with neoplastic mast cells PD-1 expressed by LAD2 cell line with suppressed growth following incubation with recombinant PD-L1
Kuklinski et al.	IHC on skin biopsies	Strong diffuse staining for PD-L1 in all mastocytosis cases (*n* = 16) regardless of subtype
Rabenhorst et al.	Serum PD-1, PD-L1, PD-L2, and tryptase, IF of BM and skin biopsies	Significantly higher serum PD-L1 in adults with mastocytosis (*n* = 31) compared to controlsSignificantly higher serum PD-L1 in patients with advSM (*n* = 10) compared to patients with ISM or mastocytosis limited to the skin (*n* = 21)
Hatch et al.	IHC on BM, skin, spleen, and LN biopsies, flow cytometry on patient samples and mast cell lines, multicolor IF on patient samples	PD-L1 expressing cells increased in number in SM and CM (*n* = 55) but not in other reactive or neoplastic bone marrows PD-L1 staining confirmed by flow cytometry PD-1 limited to a subset of CM cases

Abbreviations: IHC: immunohistochemistry, RT-PCR: reverse transcriptase polymerase chain reaction, IF: immunofluorescence, BM: bone marrow, LN: lymph node, advSM: advanced systemic mastocytosis, ISM: indolent systemic mastocytosis, CM: cutaneous mastocytosis.

## Abbreviations

PD-1	programmed death 1
CM	cutaneous mastocytosis
SM	systemic mastocytosis
ISM	indolent systemic mastocytosis
ASM	aggressive systemic mastocytosis
advSM	advanced systemic mastocytosis
SM-AHN	systemic mastocytosis with associated hematologic neoplasm
IHC	immunohistochemistry
RT-PCR	reverse transcriptase polymerase chain reaction
IF	immunofluorescence
BM	bone marrow
LN	lymph node
